# A tale of two belongings: social and academic belonging differentially shape academic and psychological outcomes among university students

**DOI:** 10.3389/fpsyg.2024.1394588

**Published:** 2025-02-12

**Authors:** Smaranda Ioana Lawrie, Delwin B. Carter, Karen Nylund-Gibson, Heejung S. Kim

**Affiliations:** ^1^Department of Psychology, Providence College, Providence, RI, United States; ^2^Department of Education, University of California, Santa Barbara, Santa Barbara, CA, United States; ^3^Department of Psychological and Brain Sciences, University of California, Santa Barbara, Santa Barbara, CA, United States; ^4^Department of Psychology, Ewha Womans University, Seoul, Republic of Korea

**Keywords:** academic belonging, first-generation students, school belonging, social belonging, latent class analysis

## Abstract

The benefits of belonging in academic settings are well established; however, past empirical research has for the most part conflated academic and social belonging. This study utilized latent class analysis (LCA) with a sample of undergraduates (*N* = 837) to determine whether distinct classes or profiles of belonging exist on a college campus and whether class membership predicts academic and psychological outcomes. Four distinct belonging classes emerged: *High Social*, *High Academic* belonging (35%), *Low Social*, *High Academic* belonging (15%), *High Social*, *Low Academic* belonging (38%), and *Low Social*, *Low Academic* belonging (12%). The results show that belonging classes play different roles. For academic outcomes (GPA), academic belonging was important, but not social belonging. For psychological outcomes (stress and self-esteem), both academic and social belonging mattered but academic belonging mattered more. These findings demonstrate that investigating the distinctive roles of academic and social belonging is a fruitful theoretical and applied endeavor.

## Introduction

The American system of higher education is far from equitable. Students of color and students from lower socioeconomic backgrounds perform worse academically and are less likely to graduate. Data from the National Center for Education Statistics (2020) shows that roughly 48% of European-American students enrolled at 4-year institutions graduate within 4 years, whereas only 24% of Black students and 34% of Latino/a students graduate within the same time frame. Building on school reforms that focus on academic skills or providing opportunities and resources, many social psychological intervention studies have been conducted to improve the experiences of disadvantaged students (Walton, 2014; Walton and Wilson, 2018). Among these efforts, one psychological construct that has received much attention is the *sense of school belonging*. Interventions designed to foster belonging have been effective in increasing subjective and objective measures of school achievement such as happiness, retention, and grade point average (GPA) (Cohen and Garcia, 2008; Walton et al., 2015; Walton and Cohen, 2011).

School belonging, defined as the extent to which students feel personally accepted, respected, included, and supported by others (Goodenow and Grady, 1993), is a multidimensional concept that extends beyond specific interpersonal relationships within the school context. Different facets, or types of belonging, contribute to an overall sense of fit in school. Classic theories of school persistence (Bean, 1980; Tinto, 1993) have distinguished between *academic belonging*, a student’s subjective assessment of their ability to meet academic demands, and *social belonging*, a student’s subjective interpretation of the quality of their social embeddedness in school. Despite this theoretical distinction, past research has often conflated these facets of belonging, and it remains unclear how these two types of belonging independently and interactively predict school outcomes.

For the present research, we tested whether these conceptually distinct types of belonging are empirically distinguishable. We examined whether distinct groups or profiles of belonging exist and how these different profiles of belonging are associated with academic (GPA) and psychological (self-esteem and stress) outcomes. We used latent class analysis which allowed us to go beyond a simple binary comparison between social and academic belonging to examine the naturally existing classes of belonging that students exhibit and the implications of belonging to these different classes.

## Belonging in schools

Harlow (1958) wrote that “man cannot live by milk alone” (p. 677). This succinct phrase aptly summarizes decades of findings on the nature of belonging. Fulfilling the need to belong is both central to the human experience and essential for survival itself (Ainsworth, 1989; Baumeister and Leary, 1995; Bowlby, 1988). When belonging needs are thwarted, individuals suffer a panoply of dire physical and mental health outcomes (e.g., Åkerlind and Hörnquist, 1992; Cacioppo et al., 2006; Perissinotto et al., 2012). Conversely, a sense of belonging inoculates against negative outcomes and promotes thriving (e.g., Cohen and Wills, 1985; Gailliot and Baumeister, 2007; Myers and Diener, 1995).

A sense of belonging plays an essential role in the school context as well. Students at all levels achieve better academic and psychological outcomes when they experience belonging in school (Anderman and Freeman, 2004; Osterman, 2000; Pittman and Richmond, 2007; Walton and Brady, 2017; Walton and Wilson, 2018). Yeager et al. (2016), for example, considered the importance of belonging alongside a constellation of other school-relevant factors such as personality, mindset, and intelligence and found that belonging was the most important factor in determining college enrollment rates. Given the importance of belonging, interventions designed to bolster students’ sense of belonging have gained tremendous traction on campuses nationwide (Allen and Kern, 2019; Yeager and Walton, 2011). Stacking empirical evidence suggests that these interventions have benefits on student outcomes and contribute to improved life outcomes into adulthood (e.g., Brady et al., 2016; Kenthirarajah and Walton, 2015; Walton, 2014; Walton and Wilson, 2018).

Past research typically has employed experimental manipulations that combine different types of school belonging. For instance, a popular experimental manipulation, aimed at teaching neophyte students that belonging is problematic for most students at the beginning of school but improves with time, exposes first-year students to stories from multiple upper-division students. Upper-division students describe initial struggles with social belonging whereas others outline struggles with academic belonging (e.g., Walton and Cohen, 2011; Yeager et al., 2016). Likewise, school belonging scales (e.g., The Psychological Sense of School Membership; Goodenow, 1993) include questions relating to both social and academic facets of belonging.

Past research has found that, to an extent, *both* academic and social belonging play an important role. Research shows that students with low academic belonging, who do not feel like they have ability to succeed, are more likely to make choices that undermine their success. According to the expectancy-value theory (Atkinson, 1964; Muenks et al., 2018; Wigfield et al., 2016), if students have low expectations for their ability to fit in academically, they will lack motivation (Berndt and Miller, 1990; Trautwein et al., 2006). And research on the imposter syndrome has found that even high achieving students who worry about academic belonging suffer negative consequences, despite above-average abilities (Clance and Imes, 1978; Parkman, 2019).

Literature similarly points to the importance of social belonging in predicting positive school outcomes. Social belonging is associated with mental health (Cohen and Wills, 1985), trust (Hillebrandt et al., 2011), self-esteem (Gailliot and Baumeister, 2007; Leary et al., 1998), happiness (Myers and Diener, 1995), and meaning (Baumeister, 1991; Lambert et al., 2013), which all have downstream implications for other school outcomes. Conversely, social exclusion, which results in a lowered sense of belonging, directly reduces reasoning abilities. In one set of studies, for instance, socially excluded participants obtained lower intelligence scores and showed impairments in reading comprehension (Baumeister et al., 2005).

Although it is reasonable to expect that both types of belonging matter in a college setting, several questions remain unanswered. First, it is unclear if students experience these two types of belonging separately or as inextricably intertwined. Are students who are high in one type of belonging necessarily high in the other type of belonging (or alternatively, low/low) or is it possible that some students are low in one type of belonging and high in the other (e.g., high academic/low social). Based on previous theorizing (e.g., Tinto, 1993), the expectation would be that students do experience these types of belonging as distinct and therefore might logically feel high belonging in the social domain and low belonging in the academic domain (or vice versa). It is also unclear how these two types of belonging, or the combination of the two, bring about outcomes such as academic performance and psychological well-being. It is possible that both types of belonging are necessary for both academic and psychological outcomes, or it could be that one type of belonging has a stronger impact on both outcomes. Alternatively, it might be that academic belonging is more important for academic outcomes, and social belonging is more critical for psychological outcomes. Resolving these mysteries is especially important from an applied perspective.

## Method

### Research design

The present study employed a correlational design to gauge the naturally occurring subjective experiences of belonging among students. A survey consisting of validated measures of subjective sense of social and academic belonging as well as psychological outcomes (self-esteem and stress) was administered at a large public university. An objective measure of academic achievement (i.e., GPA) was obtained from the registrar. All research was approved by the IRB Board at the university where the study was conducted.

Latent class analysis (LCA) was used to group students into latent classes based on response patterns to a set of indicator variables (i.e., questions about social and academic belonging). The LCA approach was used to establish the number and type of combinations of social and academic belonging (i.e., belonging classes) that exist naturally in the student population. We expected four classes would emerge based on different combinations of high and low social and academic belonging, supporting the idea that these two types of belonging are distinct. A finite mixture modeling approach provided information about how the different types of belonging are subjectively experienced by students, which is otherwise obscured by the typical additive approach to the measurement of belonging. Specifically, our LCA approach helped illustrate how social and academic belonging may work together or separately to influence school outcomes. After classes were determined, exploratory analyses were performed to test how class membership predicts GPA, self-esteem, and stress. Finally, we investigated how demographic characteristics including college generational status and ethnicity predict group membership.

### Participants

Participants were 837 students recruited in three ways: (1) subject pools, (2) email from registrar, and (3) recruitment posters. The average age of participants was 18.90 (SD = 1.39) and 28.4% of the sample identified as female while 71.4% of the sample identified as male. All data collection preceded the COVID-19 pandemic. Students received course credit or $10 as compensation. Students with at least one parent with a college degree were labeled “continuing generation” (*n* = 437). All other students were considered first-generation (*n* = 400) (Stephens et al., 2014). Previous simulation studies suggest that a sample size of 300 is sufficient to achieve adequate statistical power (Nylund-Gibson and Choi, 2018). See Supplementary material for additional sample characteristics (e.g., gender, etc.).

### Measures

#### Latent class indicators

##### Social belonging

Social belonging was measured using four items from Walton and Cohen’s (2007) Sense of Social and Academic Fit Scale. Participants indicated the extent to which they agree with statements using a 7-point scale (1 = *strongly disagree* to 7 = *strongly agree*, e.g., “*People at [college] like me*”). Two items were reverse-coded. To use items in the LCA context, the 7-item Likert categories were trichotomized. Responses of 1, 2, or 3 were coded as 1 = “*disagree*,” responses of 4 were coded as 2 = “*neutral*,” and responses of 5, 6, or 7 were coded as 3 = “*agree*.”

##### Academic belonging

Academic belonging was measured using four items adapted from Lewis and Hodges’s (2015) Ability Uncertainty Scale. The original scale was designed to measure perceived ability to succeed in specific majors, but we adapted items to gauge perceived ability to succeed in college more generally. Participants were asked the extent to which they agree with statements on a 7-point scale (1 = *strongly disagree* to 7 = *strongly agree*, e.g., “*I often wonder if I have what it takes to succeed at [college]*”). Two items were reverse-coded and Likert categories were trichotomized.

##### Predictor variables

Generational status (i.e., *first*- vs. *continuing-generation*) and ethnicity^1^ were included as covariates to see if they predict class membership.

#### Outcome variables (distal variable)

##### GPA

Cumulative GPA was obtained from the registrar.

##### Self esteem

Self-esteem was measured using the 10-item Rosenberg (1965) Self-Esteem Scale. Participants were asked how much they agreed with statements on 7-point scale (1 = *strongly disagree* to 7 = *strongly agree*, e.g., “*On the whole I am satisfied with myself*”). Five items were reverse coded (*α* = 90).

##### Stress

Stress was measured using the 10-item Perceived Stress Scale (Cohen et al., 1994). Participants were asked how often they felt a certain way in the past month on a 7-point scale (1 = *never* to 7 = *very often*, e.g., “*In the last month*, *how often have you felt nervous and stressed*?”). Four items were reverse coded (*α* = 70).

## Analytic overview

Mixture modeling was performed using Mplus, Version 8.1 (Muthén and Muthén, 1998). Analysis began with class enumeration and was followed by the addition of predictors and outcomes.

### Class enumeration

LCA using full information maximum likelihood (FIML; Rubin, 1987) was used to determine the number of underlying latent classes. Random starts were used to verify that the solution converged on the global rather than a local maximum. Six fit statistics were analyzed (Nylund et al., 2007; Masyn, 2013). The information criteria tests were the approximate weight of evidence (AWE; Banfield and Raftery, 1993), the constant Akaike information criterion (CAIC; Bozdogan, 1987), the Bayesian information criterion (BIC; Schwarz, 1978), and the sample size adjusted Bayesian information criterion (SABIC; Sclove, 1987). For information criteria, the optimal solution is indicated when the value reaches its lowest point or when the change in value becomes unreasonably small. The likelihood ratio tests compare two subsequent models (i.e., the *K* class and *K* − 1 class models). When the *K* class model compared to the *K* − 1 class model results in a non-significant *p*-value, this indicates that model fit is not an improvement over the *K* − 1 model (Nylund-Gibson and Choi, 2018). LRT tests considered consisted of the bootstrapped likelihood ratio test (BLRT; McLachlan and Peel, 2000) and the Vuong–Lo–Mendell–Rubin adjusted likelihood ratio test (VLMR-LRT; Lo et al., 2001). Class homogeneity, class separation, meaningful interpretation of individual classes, and substantive checking were also considered. Entropy was examined to check classification accuracy (Nylund-Gibson and Choi, 2018).

### Manual 3-step

**Figure 1:**
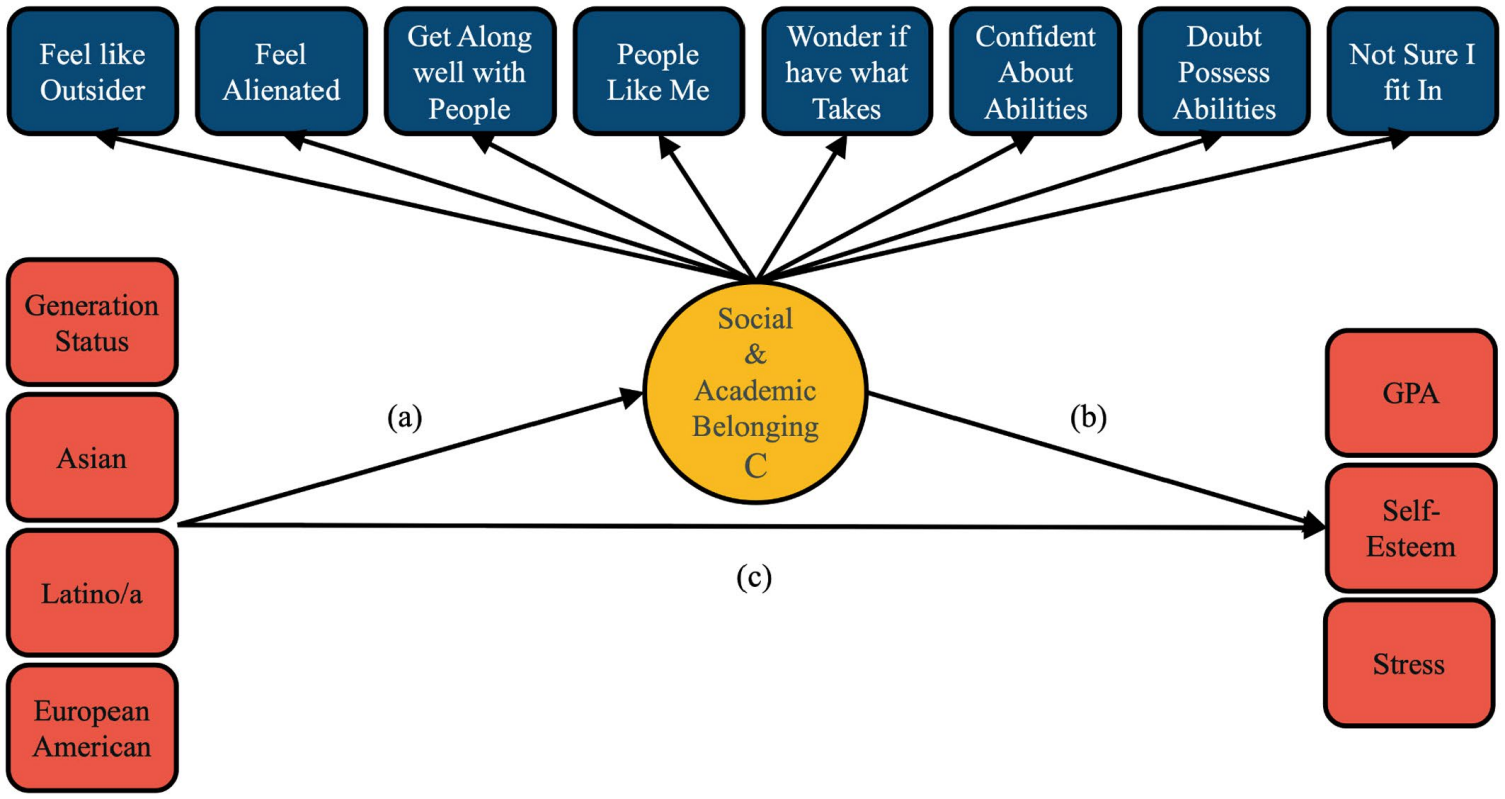
Conceptual model of the social and academic belonging mixture model. Path a = multinomial logistic regression path from the latent class variable to the covariates. Path b = regression of the distal outcomes on the latent class variable. Path c = regression of the distal outcomes on the covariates as a control measure.

Upon selection of a final class solution, auxiliary variables including the covariate (*x*) variables (ethnicity and generation) and outcome (*y*) variables (GPA, self-esteem, and stress) were considered using the manual 3-step approach. This approach permits for simultaneous modeling of both covariates (i.e., predictor variables, *x*) and outcomes (i.e., *y*) (Asparouhov and Muthén, 2014) and prevents potential shifting of latent classes (Nylund-Gibson et al., 2014; Vermunt, 2010). Thus, outcomes (*y*) were regressed onto the latent class variable *C*, which was regressed onto the covariates (*x*) by multinomial logistic regression (*x* ⟶ *C* ⟶ *y*; see Figure 1).

For outcome variables, means were estimated for each class and then subjected to an omnibus Wald test (similar to an ANOVA; Asparouhov and Muthén, 2007). Outcome variables resulting with a significant Wald test were then assessed for effect size (LTB-*ω*; Lanza et al., 2013), which allowed us to determine the strength of association of each outcome independently with the latent class variable. The LTB-*ω* coefficient is expressed in Cohen’s (1992) *d* metric (i.e., *d* = 0.20 and *d* = 0.80, large effect). The LTB-*ω* provides valuable information to mixture modeling scholarship but is relatively underused (Carter, 2022). Finally, outcome means differences by class were examined for each outcome that passed the Wald test (i.e., GPA, self-esteem, and stress). Cohen’s *d* effect sizes with their 95% confidence intervals are included for significant results.

To reduce bias in the estimates of interest (i.e., path a and b; see Figure 1), the direct relationship between outcome variables (*y*) and predictor (covariate) variables (*x*) were estimated (*x* ⟶ *y*; i.e., path c, see Figure 1) to control for their relationship. Results for the control variables can be found in Supplementary material.

## Results

All materials can be found at https://osf.io/6ykwa/?view_only=bf3ded8e6dd5476e9f88c1fcaac024a7.

### Descriptive statistics

See Supplementary material for the eight belonging latent class indicators, the covariates, and the three distal/outcome variables.

### Belonging LCA

Class enumeration was conducted on the four social and four academic belonging indicators beginning with a 1-class solution and up to a 6-class solution (see Supplementary Table S3). The BIC and CAIC supported the 4-class solution. The AWE had the lowest value at class 3, and the SABIC had the lowest value at class 6. The VLMR-LRT became nonsignificant at the 5th class, also supporting a 4-class solution. The BLRT did not become nonsignificant so did not support any solution. Because the BIC, CAIC, and the VLMR-LRT all supported the 4-class solution, it was chosen as the best-fitting model. Entropy of the 4-class solution was 0.812, indicating excellent classification accuracy. See Supplementary material for fit statistics for class enumeration.

**Figure 2:**
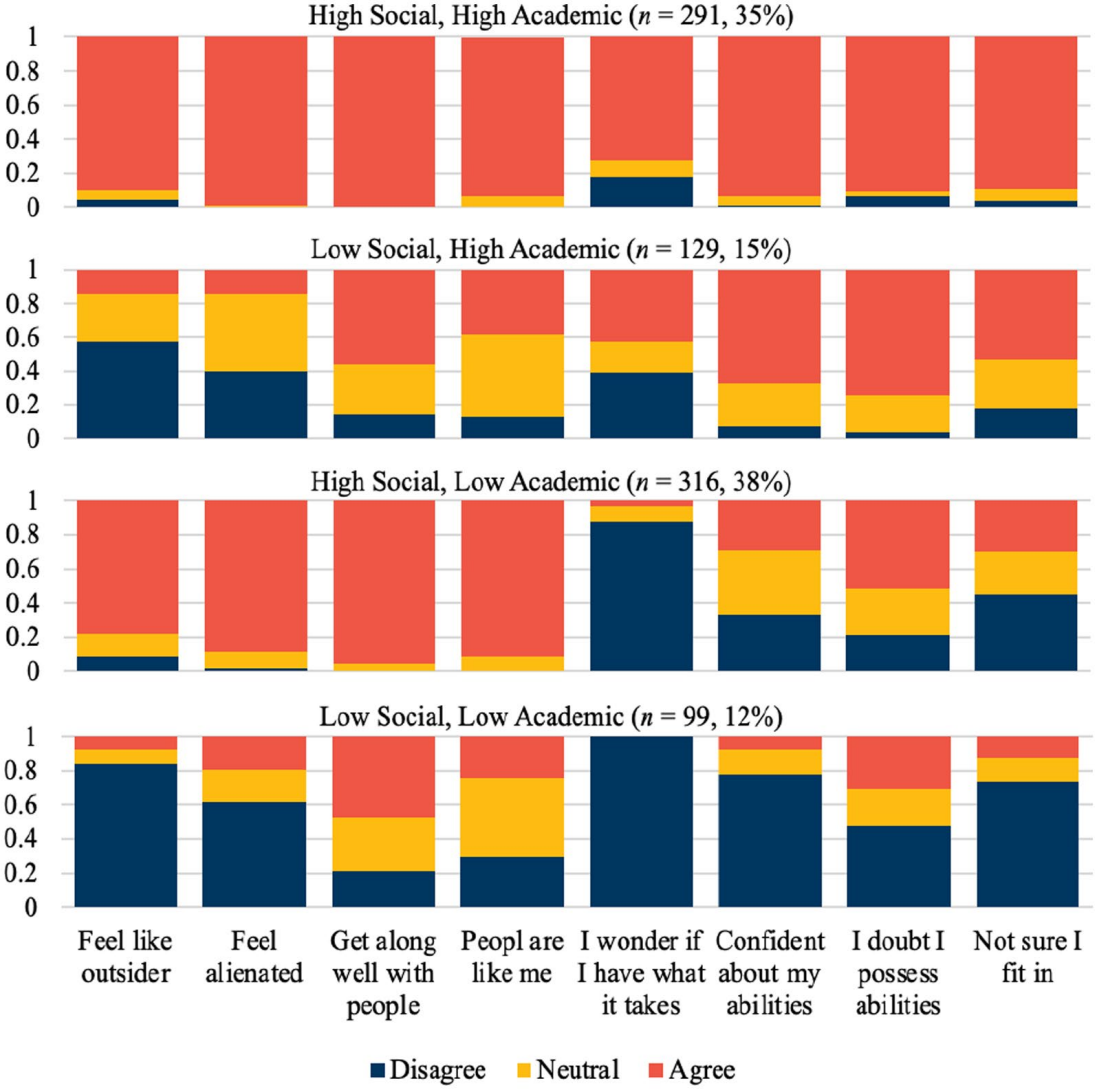
Probability plots of the four-class model.

The four belonging classes were: (1) *High Social*, *High Academic*, (2) *Low Social*, *High Academic*, (3) *High Social*, *Low Academic*, and (4) *Low Social*, *Low Academic*. The largest class was the *High Social*, *Low Academic* class, which characterized 38% of students (*n* = 316). The second largest class was the *High Social*, *High Academic* class, which characterized 35% of students (*n* = 291). The *Low Social*, *High Academic* and *Low Social*, *Low Academic* classes characterized 15% (*n* = 129) and 12% (*n* = 99) of the sample, respectively. These results show that students do not simply have high or low belonging, rather unique profiles of belonging exist (see Figure 2).

### Covariates/predictor results

**Figure 3:**
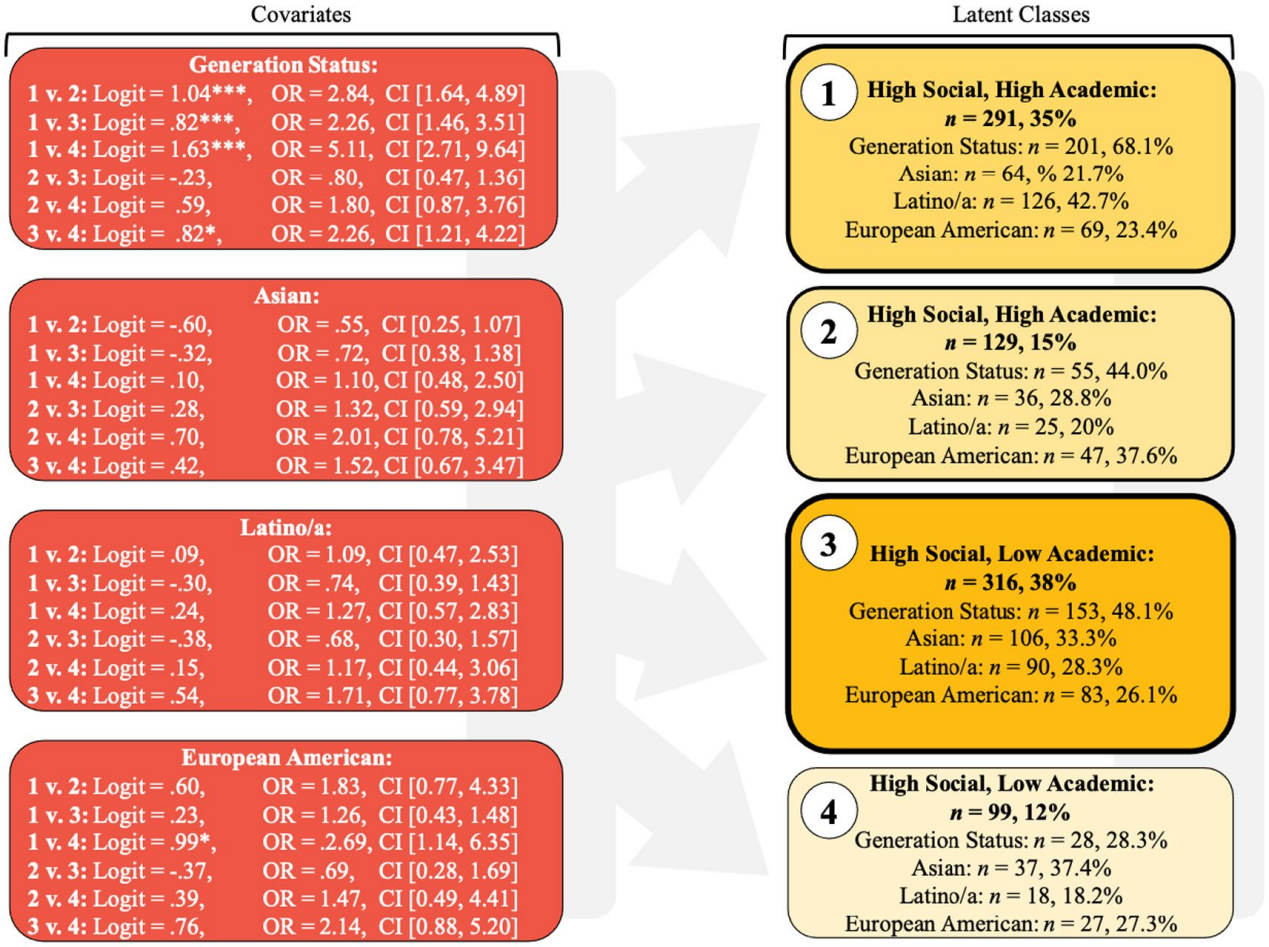
Log odds coefficients, odds ratios, *n*-size, and percentages for each covariate by class for the four-class model.

Covariate analyses revealed that continuing-generation students are more likely to fall into a class that is high on both types of belonging compared to classes high in just one form of belonging or low on both types of belonging (see Figure 3). They are likewise more likely to fall into belonging classes high in at least one form of belonging rather than in the class low on both. First-generation students make up a larger portion of the class low on both types of belonging. Although more nuanced, these findings support previous research that has found that first-generation students tend to have lower belonging compared to continuing-generation students. Compared to first-generation students, continuing-generation students in our sample were almost 5 times more likely to be in the class high on both types of belonging than the class low on both types of belonging (logit = 1.63, SE = 0.28, *p* < 0.001, OR = 5.11). Likewise, continuing-generation, compared to first-generation students, were almost 3 times more likely to be represented in the *High Social*, *High Academic* class than the *Low Social*, *High Academic* class (logit = 1.04, SE = 0.28, *p* < 0.001, OR = 2.84) and over 2 times more likely to be represented in the *High Social*, *High Academic* class than the *High Social*, *Low Academic* class (logit = 0.82, SE = 0.22, *p* < 0.001, OR = 2.26). Finally, continuing-generation students were over 2 times more likely to be in the *High Social*, *Low Academic* class than in the *Low Social*, *Low Academic* class (logit = 0.82, SE = 0.32, *p* = 0.011, OR = 2.26).

In terms of ethnicity, European American students, compared to non-European American students, were nearly 3 times more likely to be in the *High Social*, *High Academic* class than in the *Low Social*, *Low Academic* class (logit = 0.99, SE = 0.44, *p* = 0.024, OR = 2.69); however, there were no differences across class when comparing Latino/a vs. non-Latino/a and Asian vs. non-Asian.

### Distal/outcome variables

**Figure 4:**
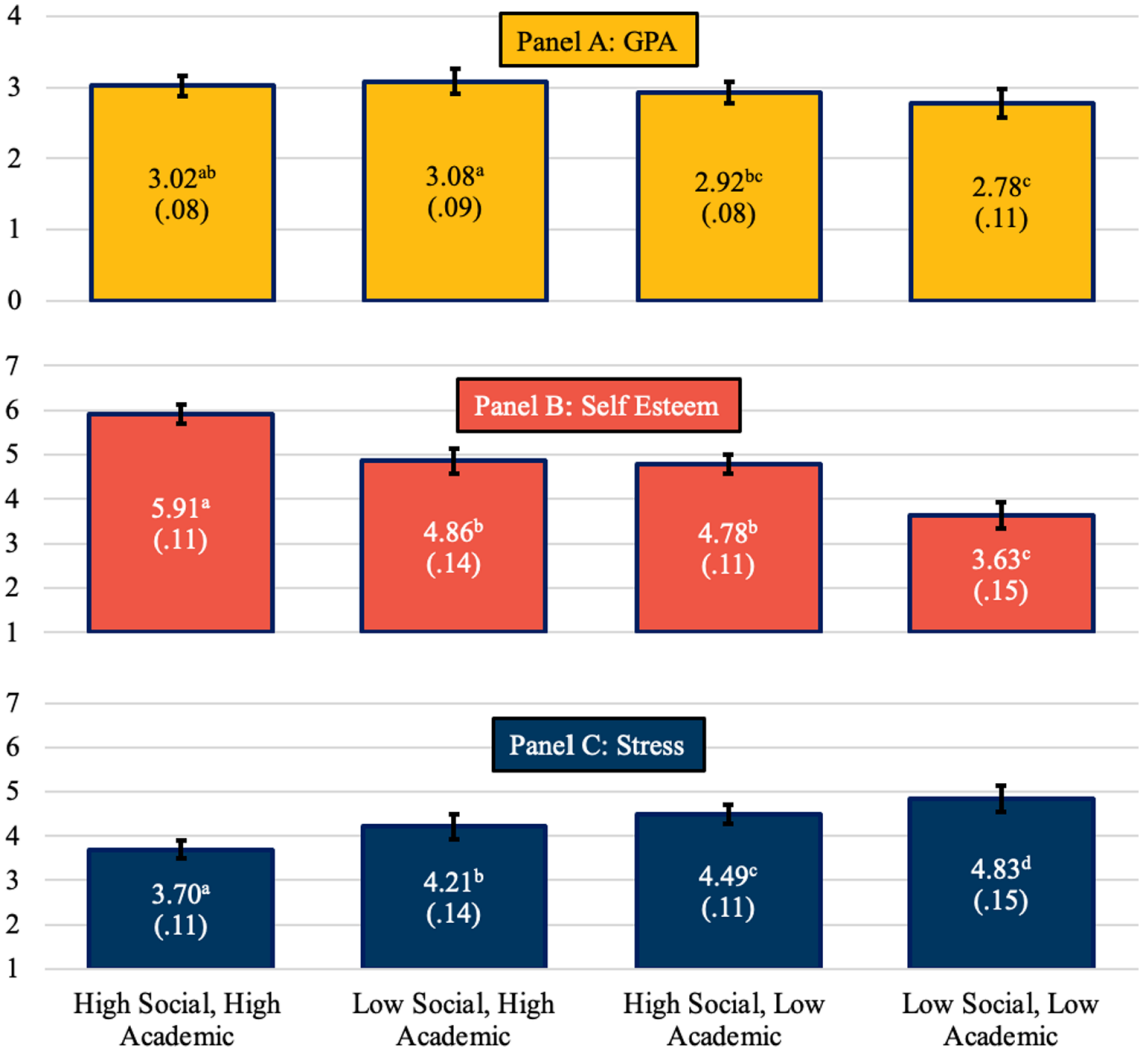
Means and standard errors of GPA in panel A and esteem and stress in panel B.

Wald tests were conducted for outcome variables. Results indicated significant differences between belonging classes on all outcome variables, thus LTB-*ω* effect sizes were calculated. Results were as follows: GPA *W* = 9.82, df = 3, *p* = 0.020, LTB-*ω* = 0.14, self-esteem *W* = 304.71, df = 3, *p* < 0.001, LTB-*ω* = 0.76, and stress *W* = 170.54, df = 3, *p* < 0.001, LTB-*ω* = 0.41. These results indicate that self-esteem is most strongly associated with the latent class variable, followed by stress and GPA. In other words, class membership significantly predicts all three outcome variables but has the strongest implications for self-esteem, then stress, then GPA. Follow-up pairwise comparison analyses were conducted. Significant results are outlined below (see Figure 4). For all comparisons, see Supplementary material.

### GPA

The GPA of students in the *High Social*, *High Academic* class was significantly higher than GPA of students in the *Low Social*, *Low Academic* class (*M*_DIFF_ = 0.24, SE = 0.10, *p* = 0.01; *d* = 0.45, 95% CI = 0.22, 0.68). Likewise, GPA of students in the *Low Social*, *High Academic* class was significantly higher than GPA of students in the *High Social*, *Low Academic* class (*M*_DIFF_ = 0.18, SE = 0.08, *p* = 0.038; *d* = 0.33, 95% CI = 0.13, 0.54) and students in the *Low Social*, *Low Academic* class (*M*_DIFF_ = 0.31, SE = 0.11, *p* = 0.005; *d* = 0.56, 95% CI = 0.29, 0.83). There were no other significant results across the classes for GPA. Effect sizes for GPA ranged from small to medium size [i.e., from ¼ to ½ of a standard deviation (STD)]. Taken together, the results show that the added value of social belonging is minimal, but the added value of academic belonging is significant.

### Self-esteem

Students reported the highest self-esteem in the *High Social*, *High Academic* class compared to students in the *Low Social*, *High Academic* class (*M*_DIFF_ = 1.05, SE = 0.13, *p* < 0.001; *d* = 1.19, 95% CI = 0.96, 1.41), the *High Social*, *Low Academic* class (*M*_DIFF_ = 1.14, SE = 0.10, *p* < 0.001; *d* = 1.23, 95% CI = 1.06, 1.40), and the *Low Social*, *Low Academic* class (*M*_DIFF_ = 2.29, SE = 0.10, *p* < 0.001; *d* = 2.73, 95% CI = 2.42, 3.02). The *Low Social*, *High Academic* class and *High Social*, *Low Academic* class were both significantly higher than the *Low Social*, *Low Academic* class (*M*_DIFF_ = 1.23, SE = 0.18, *p* < 0.001; *d* = 1.19, 95% CI = 0.90, 1.47 and *M*_DIFF_ = 1.15, SE = 0.15, *p* < 0.001; *d* = 1.13, 95% CI = 0.89, 1.37) respectively. There was no significant difference between the *Low Social*, *High Academic* and *High Social*, *Low Academic* class. Effect sizes for self-esteem differences across the classes were large and ranged from over 1 STD to nearly 3 STDs.

Results suggest that in terms of self-esteem, having a sense of *both* academic and social belonging results in the best outcomes, and having a low sense of *both* results in the worst outcomes. However, either a sense of academic or social belonging, even when experienced independently of the other type of belonging, provides at least a partial buffering effect, resulting in intermediate self-esteem values.

### Stress

Stress was significantly different across all comparisons. Stress scores for students was higher in the *Low Social*, *Low Academic* class compared to students in the *High Social*, *High Academic* class (*M*_DIFF_ = 1.14, SE = 0.10, *p* < 0.001; *d* = 1.67, 95% CI = 1.41, 1.77), the *Low social*, *High Academic* class (*M*_DIFF_ = 0.62, SE = 0.12, *p* < 0.001; *d* = 0.83, 95% CI = 0.56, 0.97), and the *High Social*, *Low Academic* class (*M*_DIFF_ = 0.35, SE = 0.10, *p* < 0.001; *d* = 0.48, 95% CI = 0.25, 0.54). Stress was significantly higher for students in the *High Social*, *Low Academic* class compared to students in the *Low Social*, *High Academic* class (*M*_DIFF_ = 0.27, SE = 0.10, *p* = 0.009; *d* = 0.71, 95% CI = 0.49, 0.92) and those in the *High Social*, *High Academic* class (*M*_DIFF_ = 0.79, SE = 0.08, *p* < 0.001; *d* = 1.10, 95% CI = 0.93, 1.07). Finally, stress scores were significantly higher for students in the *Low Social*, *High Academic* class when compared to students’ scores in the *High Social*, *High Academic* class (*M*_DIFF_ = 0.52, SE = 0.10, *p* < 0.001; *d* = 0.71, 95% CI = 0.49, 0.62). Effect sizes across the classes ranged from medium to large with differences of ½ to over 1 STD.

Taken together, unsurprisingly, experiencing high levels of *both* types of belonging results in the best outcomes, and experiencing low levels of both types of belonging results in the worst outcomes. However, there is also a significant unexpected difference between the mixed classes. Academic belonging seems to be more important than social belonging; students with high social belonging and low academic belonging experience *more* stress than students with low social belonging and high academic belonging.

## Discussion

The present research examined whether social and academic belonging are empirically distinguishable and whether either of these types of belonging or a combination of the two has implications for academic (i.e., GPA) and psychological (i.e., self-esteem and stress) outcomes. Overall, we found four distinct classes of belonging. Students do not simply experience high belonging on one end of a pole or low belonging on the other end; rather, unique combinations of social and academic belonging are experienced. Notably, however, more students overall fall into classes with low academic belonging compared to classes with low social belonging (27% vs. 50%).

Exploratory analyses revealed interesting patterns of results. For example, it is noteworthy that generational status (i.e., first- vs. continuing-generation) predicted group membership more strongly than ethnicity did. As socioeconomic status (SES) and ethnicity are often confounding factors, the current results may shed some light on the specific roles of each. Moreover, these results are a first step in establishing that academic and social belonging are experienced by students in different combinations, and that they do not necessarily function the same way in terms of student outcomes. Class membership had important implications for all three student outcomes, and effects were especially strong for subjective measures (i.e., self-esteem and stress). In terms of GPA, academic belonging was clearly important but social belonging was not. In terms of psychological outcomes, although social belonging is clearly important, the current results suggest that academic belonging might be more important than social belonging in terms of both objective and subjective outcomes. This is particularly notable given that fewer students experience high academic belonging. This may be because a university is first and foremost an academic institution. This research draws attention to the important role of context and person-environment fit; schools are different from work and family contexts in that, ultimately, they are institutions designed to teach academic subjects and therefore academic and social belonging may function differently here than in other contexts.

### Limitations

Like all studies, this research is not without limitations. Our sample was culled from a specific college environment, a competitive minority-serving institution. It is possible that worries about academic belonging were more salient in our findings because of the high academic caliber of the institution. It is equally possible that fewer students experience diminished social belonging in the current context because of the diversity present on campus. It is important to replicate this research on campuses that differ in academic rigor and diversity.

It is also possible that academic belonging was more important in the current findings because we measured social belonging, as much previous research has done, in a very general sense (feelings of social belonging at an institution). Research has found that a social relationship with just one teacher has benefits for minority students (Gehlbach et al., 2016) and that even arbitrary minimal connections (e.g., sharing a birthday) can provide benefits (Walton et al., 2012). It is possible that students who experience low social belonging on a college campus may suffer less dire consequences if they have several (or even one) solid relationships. Future research is necessary to investigate the potential buffering effect of specific relationships.

### Conclusion

This research is an early step toward a more nuanced understanding of the interplay between academic and social belonging. Ultimately, a finer-grained understanding of different types of belonging could lead to interventions specifically targeted for particular groups of students and their specific needs. This is important as school administrators, who are already stretched too thin, often struggle to work out how to transfer research findings into the best day-to-day practices.

The current research specifically suggests that the greatest number of students would benefit most from interventions geared towards building academic belonging. A number of interventions in which older students share their college-related struggles and successes with incoming students have been shown to be effective on different university campuses [e.g., Difference-Education intervention (Stephens et al., 2014; Townsend et al., 2019) or the Social-Belonging intervention (Walton et al., 2017)]. The current findings suggest that in using this type of approach, it might be useful to have stories focus to a greater extent on academic struggles and school challenges. In conjunction, it might be beneficial to incorporate a growth mindset intervention that highlights for students how failure and struggle are part of everyone’s learning story (e.g., Broda et al., 2018). This type of approach will most directly benefit the 50% of students who struggle with academic belonging but is unlikely to take anything away from students who belong to other groups (e.g., Walton et al., 2023).

## Data Availability

The datasets presented in this study can be found in online repositories. The names of the repository/repositories and accession number(s) can be found at: https://osf.io/6ykwa/?view_only=bf3ded8e6dd5476e9f88c1fcaac024a7.

## Footnotes

^1^The three ethnic groups utilized for covariate analyses were selected based on the fact that in the sample of participants, they were the largest groups numerically. Although we realize this is not an ideal way to deal with ethnic groups, we did the best we could given the large sample-size requirements for LCA.

## References

[ref1] Ainsworth M. S. (1989). Attachments beyond infancy. Am. Psychol. 44, 709–716. doi: 10.1037/0003-066X.44.4.709, PMID: 2729745

[ref2] Åkerlind I. Hörnquist J. O. (1992). Loneliness and alcohol abuse: a review of evidences of an interplay. Soc. Sci. Med. 34, 405–414. doi: 10.1016/0277-9536(92)90300-f, PMID: 1566121

[ref3] Allen K.-A. Kern P. (2019). Boosting school belonging in adolescents: interventions for teachers and mental health professionals. London: Routledge.

[ref4] Anderman L. H. Freeman T. M. (2004). “Students’ sense of belonging in school” in Motivating students, improving schools: the legacy of Carol Midgley. Advances in motivation and achievement. eds. Pintrich R. Maehr M. L. (Amsterdam: JAI Press), 27–63.

[ref5] Asparouhov T. Muthén B. O. (2007). Wald test of mean equality for potential latent class predictors in mixture modeling. Available at: https://www.statmodel.com/download/MeanTest1.pdf (Accessed November 16, 2007).

[ref6] Asparouhov T. Muthén B. (2014). Auxiliary variables in mixture modeling: three-step approaches using M*plus*. Struct. Equ. Model. Multidiscip. J. 21, 329–341. doi: 10.1080/10705511.2014.915181

[ref7] Atkinson J. W. (1964). An introduction to motivation. New York: Van Nostrand.

[ref8] Banfield J. D. Raftery A. E. (1993). Model-based Gaussian and non-Gaussian clustering. Biometrics 49, 803–821. doi: 10.2307/2532201

[ref9] Baumeister R. F. (1991). Meanings of life. New York: Guilford Press.

[ref10] Baumeister R. F. DeWall C. N. Ciarocco N. J. Twenge J. M. (2005). Social exclusion impairs self-regulation. J. Pers. Soc. Psychol. 88, 589–604. doi: 10.1037/0022-3514.88.4.589, PMID: 15796662

[ref11] Baumeister R. F. Leary M. R. (1995). The need to belong: desire for interpersonal attachments as a fundamental human motivation. Psychol. Bull. 117, 497–529. doi: 10.1037/0033-2909.117.3.497, PMID: 7777651

[ref12] Bean J. P. (1980). Dropouts and turnover: the synthesis and test of a causal model of student attrition. Res. High. Educ. 12, 155–187. doi: 10.1007/bf00976194

[ref13] Berndt T. J. Miller K. E. (1990). Expectancies, values, and achievement in junior high school. J. Educ. Psychol. 82, 319–326. doi: 10.1037/0022-0663.82.2.319

[ref14] Bowlby J. (1988). A secure base: parent-child attachment and healthy human development. New York: Basic Books.

[ref15] Bozdogan H. (1987). Model selection and Akaike’s information criterion (AIC): the general theory and its analytical extensions. Psychometrika 52, 345–370. doi: 10.1007/bf02294361

[ref16] Brady S. T. Walton G. M. Jarvis S. N. Cohen G. L. (2016). Bending the river: downstream consequences of a social-belonging intervention in the transition to college. Stanford, CA: Department of Psychology, Stanford University (Manuscript in preparation).

[ref17] Broda M. Yun J. Schneider B. Yeager D. S. Walton G. M. Diemer M. (2018). Reducing inequality in academic success for incoming college students: a randomized trial of growth mindset and belonging interventions. J. Res. Educ. Effect. 11, 317–338. doi: 10.1080/19345747.2018.1429037, PMID: 38250254 PMC10798796

[ref18] Cacioppo J. T. Hughes M. E. Waite L. J. Hawkley L. C. Thisted R. A. (2006). Loneliness as a specific risk factor for depressive symptoms: cross-sectional and longitudinal analyses. Psychol. Aging 21, 140–151. doi: 10.1037/0882-7974.21.1.140, PMID: 16594799

[ref19] Carter D. (2022). “The LTB-*ω* provides valuable information to mixture modeling scholarship but is relatively underused” in Master’s thesis (Santa Barbara: University of California).

[ref20] Clance P. R. Imes S. A. (1978). The imposter phenomenon in high achieving women: dynamics and therapeutic intervention. Psychother.: Theory Res. Pract. 15, 241–247. doi: 10.1037/h0086006

[ref21] Cohen J. (1992). Statistical power analysis. Curr. Dir. Psychol. Sci. 1, 98–101. doi: 10.1111/1467-8721.ep10768783

[ref22] Cohen G. L. Garcia J. (2008). Identity, belonging, and achievement. Curr. Dir. Psychol. Sci. 17, 365–369. doi: 10.1111/j.1467-8721.2008.00607.x

[ref23] Cohen S. Kamarck T. Mermelstein R. (1994). “Perceived stress scale” in Measuring stress: a guide for health and social scientists. eds. Cohen S. Kessler R. C. Underwood Gordon L. (Oxford: Oxford University Press), 235–283.

[ref24] Cohen S. Wills T. A. (1985). Stress, social support, and the buffering hypothesis. Psychol. Bull. 98, 310–357. doi: 10.1037/0033-2909.98.2.310 3901065

[ref26] Gailliot M. T. Baumeister R. F. (2007). Self-esteem, belongingness, and worldview validation: does belongingness exert a unique influence upon self-esteem? J. Res. Pers. 41, 327–345. doi: 10.1016/j.jrp.2006.04.004

[ref001] Gehlbach H. Brinkworth M. E. King A. M. Hsu L. M. McIntyre J. Rogers T.. (2016). Creating birds of similar feathers: Leveraging similarity to improve teacher-student relationships and academic achievement. J. Educ. Psychol. 108, 342–352. doi: 10.1037/edu0000042

[ref27] Goodenow C. (1993). The psychological sense of school membership among adolescents: scale development and educational correlates. Psychol. Sch. 30, 79–90. doi: 10.1002/1520-6807(199301)30:1<79::AID-PITS2310300113>3.0.CO;2-X, PMID: 9706331

[ref28] Goodenow C. Grady K. E. (1993). The relationship of school belonging and friends’ values to academic motivation among urban adolescent students. J. Exp. Educ. 62, 60–71. doi: 10.1080/00220973.1993.9943831

[ref29] Harlow H. F. (1958). The nature of love. Am. Psychol. 13, 673–685. doi: 10.1037/h0047884 4984312

[ref30] Hillebrandt H. Sebastian C. Blakemore S. (2011). Experimentally induced social inclusion influences behavior on trust games. Cogn. Neurosci. 2, 27–33. doi: 10.1080/17588928.2010.515020, PMID: 24168421

[ref31] Kenthirarajah D. Walton G. M. (2015). “How brief social-psychological interventions can cause enduring effects” in Emerging trends in the social and behavioral sciences. eds. Scott R. A. Kosslyn S. M. (Thousand Oaks, CA: SAGE Publications), 1–15.

[ref32] Lambert N. M. Stillman T. F. Hicks J. A. Kamble S. Baumeister R. F. Fincham F. D. (2013). To belong is to matter: sense of belonging enhances meaning in life. Personal. Soc. Psychol. Bull. 39, 1418–1427. doi: 10.1177/0146167213499186, PMID: 23950557

[ref33] Lanza S. T. Tan X. Bray B. C. (2013). Latent class analysis with distal outcomes: a flexible model-based approach. Struct. Equ. Model. Multidiscip. J. 20, 1–26. doi: 10.1080/10705511.2013.742377, PMID: 25419096 PMC4240499

[ref34] Leary M. R. Haupt A. L. Strausser K. S. Chokel J. T. (1998). Calibrating the sociometer: the relationship between interpersonal appraisals and the state self-esteem. J. Pers. Soc. Psychol. 74, 1290–1299. doi: 10.1037/0022-3514.74.5.1290, PMID: 9599444

[ref35] Lewis K. L. Hodges S. D. (2015). Expanding the concept of belonging in academic domains: development and validation of the ability uncertainty scale. Learn. Individ. Differ. 37, 197–202. doi: 10.1016/j.lindif.2014.12.002

[ref36] Lo Y. Mendell N. R. Rubin D. B. (2001). Testing the number of components in a normal mixture. Biometrika 88, 767–778. doi: 10.1093/biomet/88.3.767

[ref37] Masyn K. E. (2013). “Latent class analysis and finite mixture modeling” in The Oxford handbook of quantitative methods in psychology. Statistical analysis. ed. Little T. D. (Oxford: Oxford University Press), 551–611.

[ref38] McLachlan G. Peel D. (2000). Finite mixture modeling. New York: John Wiley & Sons.

[ref39] Muenks K. Wigfield A. Eccles J. S. (2018). I can do this! The development and calibration of children’s expectations for success and competence beliefs. Dev. Rev. 48, 24–39. doi: 10.1016/j.dr.2018.04.001

[ref40] Muthén L. K. Muthén B. O. (1998). Mplus user’s guide. 8th Edn. Los Angeles, CA: Muthén & Muthén.

[ref41] Myers D. G. Diener E. (1995). Who is happy? Psychol. Sci. 6, 10–19. doi: 10.1111/j.1467-9280.1995.tb00298.x

[ref42] National Center for Education Statistics (2020). Status and trends in the education of racial and ethnic groups. Washington, DC: National Center for Education Statistics.

[ref43] Nylund K. L. Asparouhov T. Muthén B. O. (2007). Deciding on the number of classes in latent class analysis and growth mixture modeling: a Monte Carlo simulation study. Struct. Equ. Model. Multidiscip. J. 14, 535–569. doi: 10.1080/10705510701575396

[ref44] Nylund-Gibson K. Choi A. Y. (2018). Ten frequently asked questions about latent class analysis. Transl. Issues Psychol. Sci. 4, 440–461. doi: 10.1037/tps0000176

[ref45] Nylund-Gibson K. Grimm R. Quirk M. Furlong M. (2014). A latent transition mixture model using the three-step specification. Struct. Equ. Model. 21, 439–454. doi: 10.1080/10705511.2014.915375

[ref46] Osterman K. F. (2000). Students’ need for belonging in the school community. Rev. Educ. Res. 70, 323–367. doi: 10.3102/00346543070003323

[ref47] Parkman A. (2019). The imposter phenomenon in higher education: incidence and impact. J. High. Educ. Theory Pract. 16. Available at: https://articlegateway.com/index.php/JHETP/article/view/1936

[ref48] Perissinotto C. M. Stijacic Cenzer I. Covinsky K. E. (2012). Loneliness in older persons. Arch. Intern. Med. 172, 1078–1083. doi: 10.1001/archinternmed.2012.1993, PMID: 22710744 PMC4383762

[ref49] Pittman L. D. Richmond A. (2007). Academic and psychological functioning in late adolescence: the importance of school belonging. J. Exp. Educ. 75, 270–290. doi: 10.3200/jexe.75.4.270-292

[ref50] Rosenberg M. (1965). Society and the adolescent self-image. Princeton, NJ: Princeton University Press.

[ref51] Rubin D. B. (1987). Multiple imputation for nonresponse in surveys. New York: John Wiley & Sons.

[ref53] Schwarz G. (1978). Estimating the dimension of a model. Ann. Stat. 6, 461–464. doi: 10.1214/aos/1176344136, PMID: 38281721

[ref54] Sclove S. L. (1987). Application of model-selection criteria to some problems in multivariate analysis. Psychometrika 52, 333–343. doi: 10.1007/bf02294360

[ref56] Stephens N. M. Hamedani M. G. Destin M. (2014). Closing the social-class achievement gap: a difference-education intervention improves first-generation students’ academic performance and all students’ college transition. Psychol. Sci. 25, 943–953. doi: 10.1177/0956797613518349, PMID: 24553359

[ref57] Tinto V. (1993). Leaving college: rethinking the causes and cures of student attrition. Chicago, IL: University of Chicago Press.

[ref58] Townsend S. S. Stephens N. M. Smallets S. Hamedani M. G. (2019). Empowerment through difference: an online difference-education intervention closes the social class achievement gap. Personal. Soc. Psychol. Bull. 45, 1068–1083. doi: 10.1177/0146167218804548, PMID: 30404569

[ref59] Trautwein U. Lüdtke O. Köller O. Baumert J. (2006). Self-esteem, academic self-concept, and achievement: how the learning environment moderates the dynamics of self-concept. J. Pers. Soc. Psychol. 90, 334–349. doi: 10.1037/0022-3514.90.2.334, PMID: 16536654

[ref60] Vermunt J. K. (2010). Latent class modeling with covariates: two improved three-step approaches. Polit. Anal. 18, 450–469. doi: 10.1093/pan/mpq025

[ref61] Walton G. M. (2014). The new science of wise psychological interventions. Curr. Dir. Psychol. Sci. 23, 73–82. doi: 10.1177/0963721413512856

[ref62] Walton G. M. Brady S. T. (2017). “The many questions of belonging” in Handbook of competence and motivation: theory and application. eds. Elliot A. J. Dweck C. S. Yeager D. S. (New York: Guilford Press), 272–293.

[ref63] Walton G. M. Cohen G. L. (2007). A question of belonging: race, gender, social fit, and achievement. J. Pers. Soc. Psychol. 92, 82–96. doi: 10.1037/0022-3514.92.1.82 17201544

[ref64] Walton G. M. Cohen G. L. (2011). A brief social-belonging intervention improves academic and health outcomes of minority students. Science 331, 1447–1451. doi: 10.1126/science.1198364, PMID: 21415354

[ref65] Walton G. M. Cohen G. L. Cwir D. Spencer S. J. (2012). Mere belonging: the power of social connections. J. Pers. Soc. Psychol. 102, 513–532. doi: 10.1037/a0025731, PMID: 22023711

[ref66] Walton G. M. Logel C. Peach J. M. Spencer S. J. Zanna M. P. (2015). Two brief interventions to mitigate a “chilly climate” transform women’s experience, relationships, and achievement in engineering. J. Educ. Psychol. 107, 468–485. doi: 10.1037/a0037461

[ref67] Walton G. M. Murphy M. C. Logel C. Yeager D. S. Goyer J. P. Brady S. T.. (2023). Where and with whom does a brief social-belonging intervention promote progress in college? Science 380, 499–505. doi: 10.1126/science.ade4420, PMID: 37141344

[ref68] Walton G. M. Murphy M. C. Logel C. Yeager D. S. The College Transition Collaborative (2017). “The social-belonging intervention: a guide for use and customization” in Handbook of wise interventions (New York: Guilford Press).

[ref69] Walton G. M. Wilson T. D. (2018). Wise interventions: psychological remedies for social and personal problems. Psychol. Rev. 125, 617–655. doi: 10.1037/rev0000115, PMID: 30299141

[ref70] Wigfield A. Tonks S. Klauda S. L. (2016). “Expectancy value theory” in Handbook of motivation in school. eds. Wentzel K. R. Miele D.. 2nd ed (London: Routledge), 55–74.

[ref71] Yeager D. S. Walton G. M. (2011). Social-psychological interventions in education: they’re not magic. Rev. Educ. Res. 81, 267–301. doi: 10.3102/0034654311405999

[ref72] Yeager D. S. Walton G. M. Brady S. T. Akcinar E. N. Paunesku D. Keane L.. (2016). Teaching a lay theory before college narrows achievement gaps at scale. Proc. Natl. Acad. Sci. U.S.A. 113, E3341–E3348. doi: 10.1073/pnas.152436011 27247409 PMC4914175

