# Supplementary material for: A tale of two belongings: social and academic belonging differentially shape academic and psychological outcomes among university students
Source: Front Psychol. 2025 Feb 12;15:1394588. doi: 10.3389/fpsyg.2024.1394588 (PMC11861437; doi:10.3389/fpsyg.2024.1394588)

**Supplemental Materials**

**Table S1**

*Sample Characteristics*

**
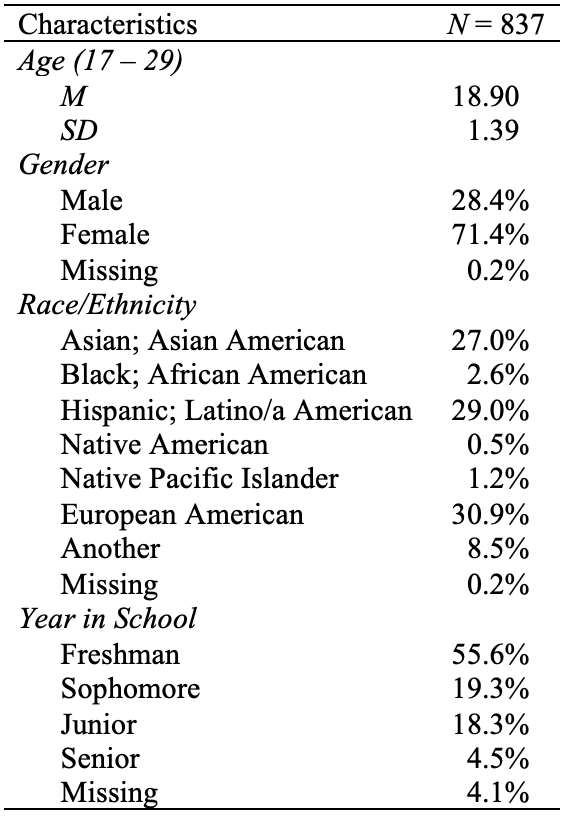
**

**Table S2**

*Descriptive Statistics for the eight belonging latent class indicators, the covariates, and the three distal/outcome variables.*


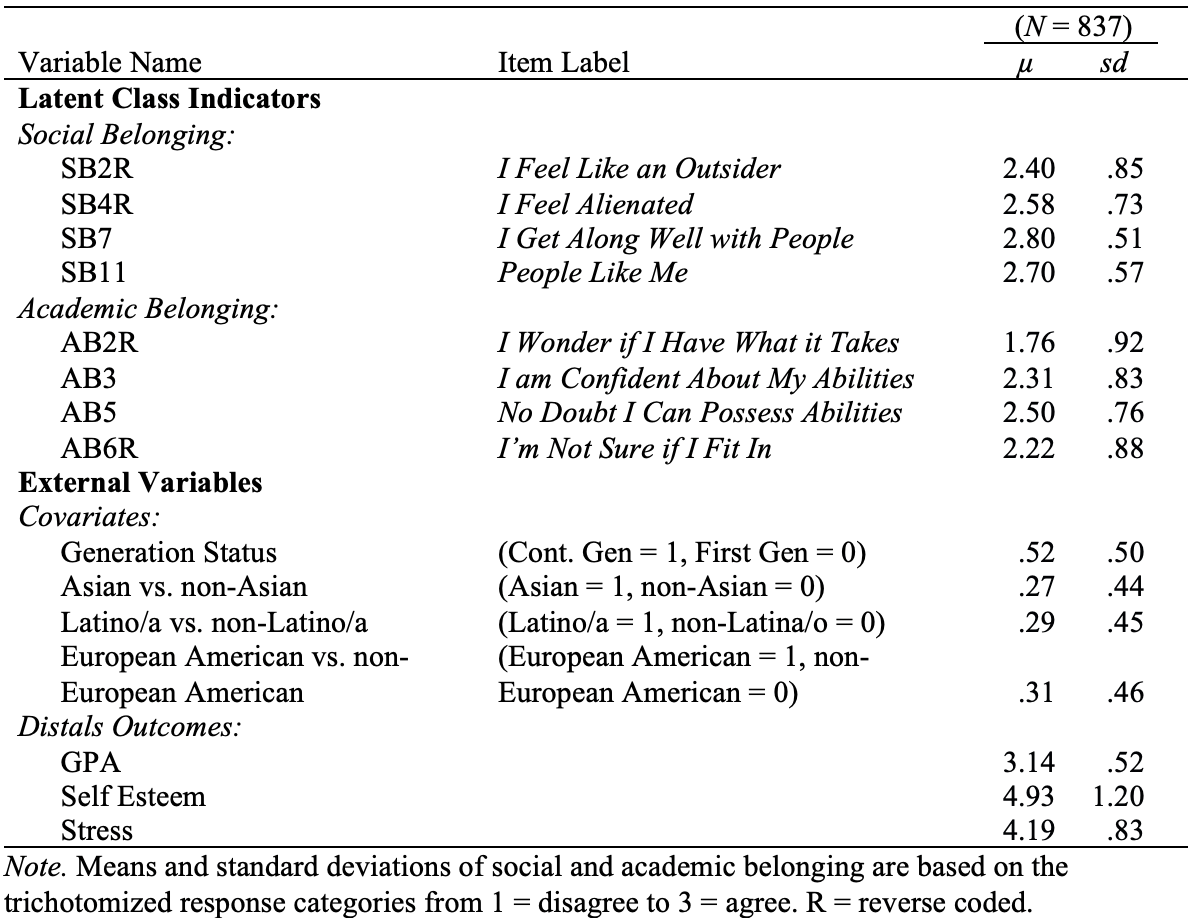


**Table S3**

*Fit Statistics for Class Enumeration*

**
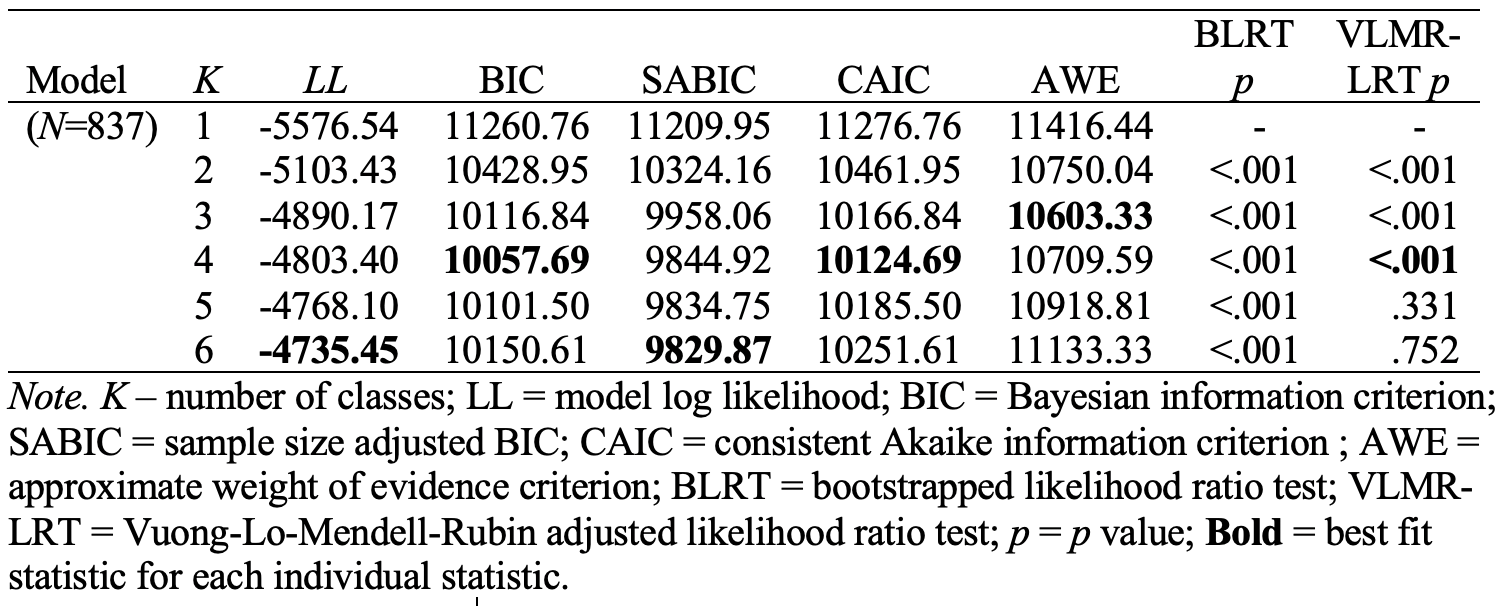
**

**Table S4**

*Regression Results for the Control Variables*

**
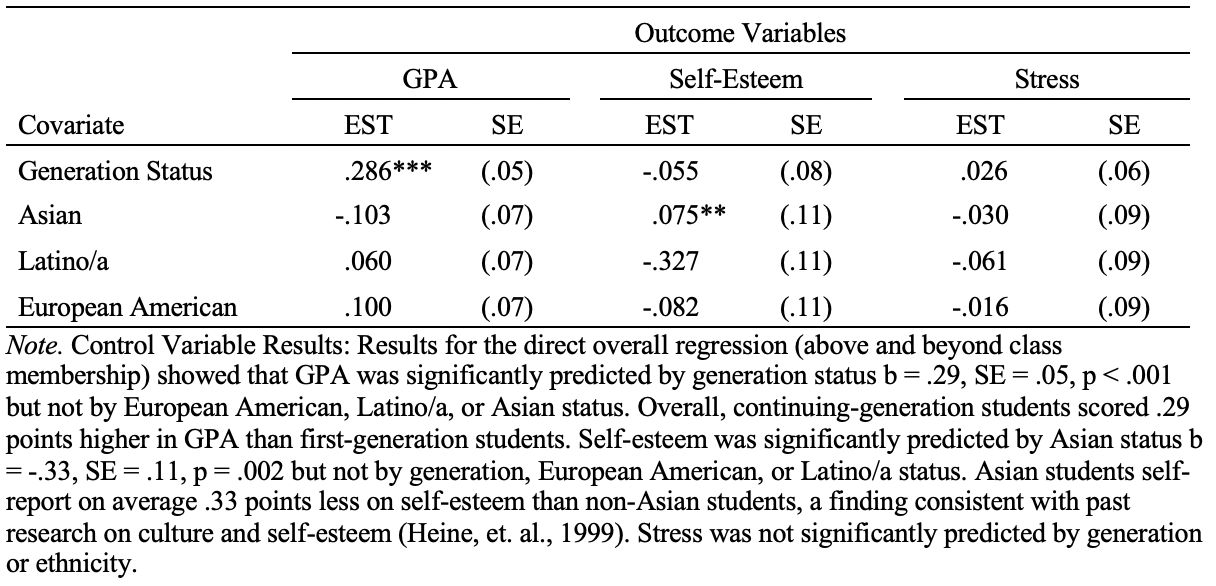
**

**Table S5**

*Percentage of Ethnicity by Class for All, First, and Continuing Generation Students*

*
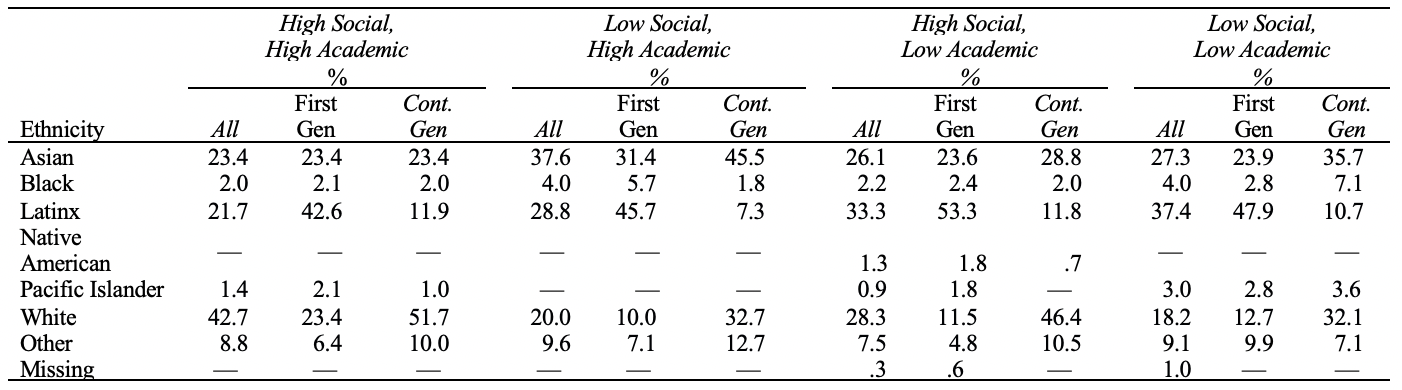
*

*.*

**Table S6**

*Outcome Means for Each Profile, Differences, and Cohen’s d for Each Comparison*


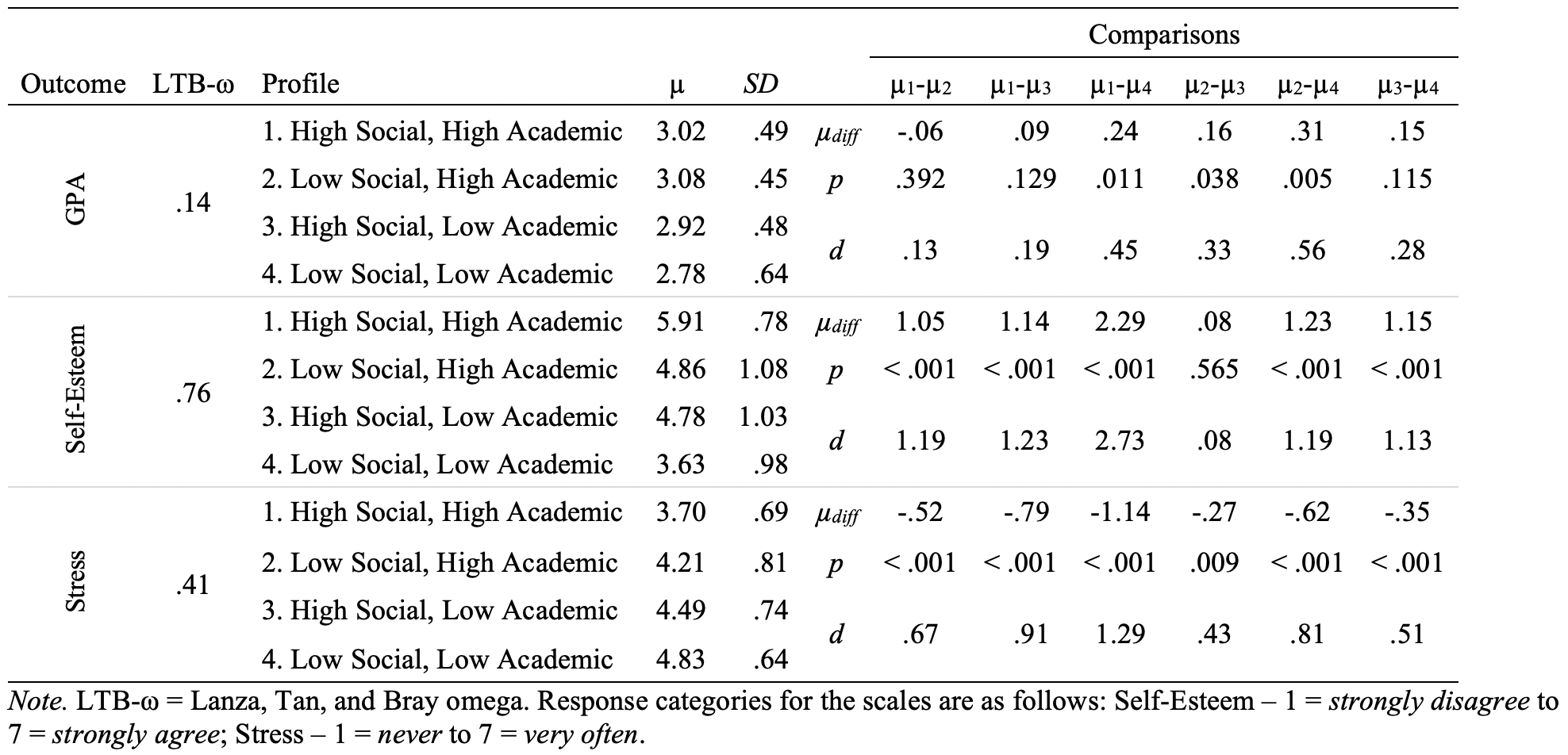

Supplement: Supplementary file 1 [file Table_1.DOCX]
